# SRPK1 and Akt Protein Kinases Phosphorylate the RS Domain of Lamin B Receptor with Distinct Specificity: A Combined Biochemical and *In Silico* Approach

**DOI:** 10.1371/journal.pone.0154198

**Published:** 2016-04-22

**Authors:** Nikolaos Voukkalis, Maria Koutroumani, Christoforos Zarkadas, Eleni Nikolakaki, Metaxia Vlassi, Thomas Giannakouros

**Affiliations:** 1 Laboratory of Biochemistry, Department of Chemistry, Aristotle University, Thessaloniki, Greece; 2 Institute of Biosciences & Applications, National Centre for Scientific Research "Demokritos", Athens, Greece; University of Minnesota, UNITED STATES

## Abstract

Activated Akt has been previously implicated in acting on RS domain-containing proteins. However, it has been questioned whether its action is direct or it is mediated by co-existing SR kinase activity. To address this issue we studied in detail the phosphorylation of Lamin B Receptor (LBR) by Akt. Using synthetic peptides and a set of recombinant proteins expressing mutants of the LBR RS domain we now demonstrate that while all serines of the RS domain represent more or less equal phosphoacceptor sites for SRPK1, Ser80 and Ser82 are mainly targeted by Akt. 3D-modeling combined with molecular dynamics (MD) simulations show that amongst short, overlapping LBR RS-containing peptides complying with the minimum Akt recognition consensus sequence, only those bearing phosphosites either at Ser80 or Ser82 are able to fit into the active site of Akt, at least as effectively as its known substrate, GSK3-β. Combined our results provide evidence that Akt kinases directly phosphorylate an RS domain-containing protein and that both the residues N-terminal the phosphosite and at position +1 are essential for Akt specificity, with the latter substrate position being compatible with the arginine residue of RS-repeats.

## Introduction

The mammalian genome encodes for more than 100 RS domain-containing proteins, almost half of which are implicated in regulating mRNA processing and the remaining half in a variety of other cellular functions [1]. Arg-Ser dipeptide repeats vary in both length and position and have been implicated in protein-protein interactions as well as in non-sequence specific interactions with RNA molecules [2, 3]. The most studied regulation of RS domain-containing proteins is through phosphorylation of serine residues within the RS domains, catalyzed mainly by the SRPK and CLK (Cdc2-like kinase) families of protein kinases. Phosphorylation induces structural changes which have been shown to modulate the interactions of RS domains in the cell [2–5].

SRPKs and CLKs phosphorylate RS domain-containing proteins with distinct specificities and mechanisms. The specificity of SRPK1 in phosphorylating Arg-Ser/Ser-Arg dipeptides is remarkable, as mutations of Ser to Thr or Arg to Lys completely abrogate phosphorylation, while CLKs exhibit a broader specificity, phosphorylating not only Ser-Arg but also Ser-Lys or Ser-Pro sites [6, 7]. SRPK1 uses a sequential and processive phosphorylation mechanism, in which the kinase adds phosphates from the C-terminal to the N-terminal end of the Arg-Ser repeats [8]. Binding of SRPK1 to its substrates is mainly mediated by conserved docking motifs, generally rich in basic residues, that conform to the consensus sequence R-X-R/K-X-X-X-R and bind to an acidic groove of SRPK1 [4, 9–11]. Interestingly, it has recently been reported that the acidic groove of SRPK1 is dispensable for Tra2β phosphorylation, implying that SRPK1 may conform to the specific RS domain architecture of some substrates, using a flexible catalytic mechanism [12]. In this respect, it was also suggested that with short RS repeats the phosphorylation mechanism becomes distributive and the protein substrate may be required to dissociate and re-bind after each round of phosphorylation [12]. Unlike SRPK1, CLK1 appears to randomly phosphorylate serines within RS domains [13].

Akt kinases have also been implicated in the signaling pathways of RS-domain containing proteins and especially of SR proteins. It has been reported that activated Akt may indirectly influence the functionality of SR proteins through modulation of the activity and/or localization of SRPK and CLK kinases [14–16]. More specifically, activated Akt was shown to interact with and promote SRPK1 (and potentially SRPK2) autophosphorylation and its subsequent translocation into the nucleus [14], to phosphorylate SRPK2 and promote its nuclear translocation [15] and to phosphorylate and alter the activity and/or stability of CLK1 and CLK2 [16–18]. Yet, given that lengthy RS repeats contain several putative Akt consensus motifs (RXRXXS/T) it has also been reported that activated Akt may directly act on SR proteins [16, 19, 20]. Two observations further strengthen the conclusion that Akt kinases themselves are phosphorylating SR proteins. First, Akt2 was found to phosphorylate several SR proteins distinct from CLK1 in response to insulin [16] and second, overexpression of different members of SRPKs and CLKs, exerted opposite effects on fibronectin alternative splicing to that observed with Akt [19]. However, the data leading to the suggestion that SR proteins might be direct substrates for activated Akt have been seriously questioned by Zhou et al. [14] on the basis of evidence showing that the described activity of immunopurified Akt to phosphorylate SR proteins originates from associated SRPKs. Furthermore, according to the data reported by Zhou et al. [14], even highly purified constitutively active Akt from a commercial source appears to contain both Akt and SR kinase activities [14].

To shed light on this discrepancy we analyzed the phosphorylation of the RS domain of LBR, a well known substrate of SRPK1 [21, 22], by SRPK1 and Akt kinases. A set of peptides containing either the entire LBR RS domain or parts of it as well as mutations of individual serine residues within this domain were employed to demonstrate that SRPK1 and Akt kinases phosphorylate distinct sites in this substrate. In addition, we used 3D-modeling followed by MD simulations of various LBR RS-containing peptides in complex with Akt2, to further investigate the interaction of Akt with this type of potential peptide-substrates. In the past, we used a similar approach to study the recognition of the LBR RS-domain by SRPK1 kinase [4]. *In silico* modeling and MD simulation studies have been extensively used in the literature to investigate recognition/interactions of peptide-substrates with various enzymes, including protein kinases [23, 24]. Combined, our *in silico* studies further support the observation that only specific serines are targeted by Akt kinases, whereas all serines of the RS domain represent more or less equal phosphoacceptor sites for SRPK1. We propose that the balance and the concerted action of RS kinases constitute a fine-tuning mechanism that modulates the activity and function of RS domain-containing proteins.

## Materials and Methods

### Construction of plasmids and expression of recombinant proteins

The pGEX-2T bacterial expression vector (Amersham Biosciences) was used to construct plasmids that encode a fragment of the N-terminal domain of turkey LBR comprising amino acids 62–92 (LBRNt(62–92)), LBRNt(62–92) missing the RS dipeptides (deletion of residues 75–84; construct termed LBRNt(62–92)ΔRS) and five mutated forms in which Ser76, Ser78, Ser80, Ser82 and Ser84 were mutated to Gly (LBRNt(62–92)S76G), Gly (LBRNt(62–92)S78G), Ala (LBRNt(62–92)S80A), Ala (LBRNt(62–92)S82A) and Ala (LBRNt(62–92)S84A), respectively. The cDNAs coding for LBRNt(62–92) and its mutant forms were amplified with PCR from the respective cDNAs of the entire N-terminal domain of chicken LBR [21] with the upstream primer 5’-CGCGGATCCCAGAGGAAAAGCCAGTCTTCCTCA-3’ and downstream primer 5’-GCGGAATTCGCCTTTTGCTGGCCGACC-3’ and ligated into the BamHI/EcoRI site of pGEX-2T. The GST fusion proteins were produced in bacteria and purified using glutathione-Sepharose beads according to the manufacturer’s instruction. Due to the very basic character of LBRNt(62–92) and mutants (theoretical pI:12.48–12.90), the solubility of the produced recombinant proteins was dramatically increased by adding 1 M NaCl to all steps of the purification procedure, except the last step, where they were recovered from the beads using the normal elution buffer, consisting of 50 mM Tris-HCl pH 8.0 and 10 mM reduced glutathione. SRPK1 was subcloned into pGEX-2T and expressed in bacteria as GST fusion protein as previously described [22]. Myristoylated HA-Akt1 and FLAG-Akt2 subcloned into the pcDNA3.1 expression vector were kindly provided by Dr. Jim Woodgett (Lunenfeld-Tanenbaum Research Institute, Toronto, Canada). Attachment of the src myristoylation signal targets Akt to the cell membrane resulting in constitutively activated kinase through an increase in its level of phosphorylation [25].

### *In vitro* kinase assays

Kinase assays were carried out in a total volume of 25 μl containing 12 mM Hepes pH 7.5, 10 mM MgCl_2_, 25 μM ATP, an amount of the appropriate substrate as indicated and 0.19 μM (0.5 μg) GST-SRPK1 or 0.07 μM (0.1 μg) Akt for 30 min at 30°C. Recombinant active Akt1 and Akt2 were purchased from Upstate Biotechnology (Catalog# 14–276, current supplier Millipore Ltd.) and Life Technologies (Catalog# PV3184), respectively. Histone H2B (Catalog No 223514) and myelin basic protein (MBP) were purchased from Boehringer Mannheim and Life Technologies, respectively. R0, R1 and R2 peptides were previously described [21]. Incorporation of radioactivity was measured by excising the radioactive bands from an SDS-PAGE gel and scintillation counting. Michaelis constant (*K*_m_) was determined from double-reciprocal plots of 1/*V* against 1/*S*, where *V* is the rate of phosphorylation and *S* is the substrate concentration. For the determination of *K*_m_ values the concentration of ATP was raised to 100 μM. Kinase activities were determined at eight concentrations of GST-LBRNt(62–92) (0.096, 0.2, 0.4, 0.82, 1.224, 1.63, 2.45 and 3.26 μM). One unit of activity is the amount of enzyme required to catalyze the transfer of 0.6 pmol phosphate to 50 pmol (1.5 μg) GST-LBRNt(62–92) per min.

### Cell culture, immunoprecipitation and pull-down assays

Human 293T cells grown on 10 cm dishes were transfected with pcDNA3.1 constructs expressing myristoylated HA-Akt1 and FLAG-Akt2 using the calcium phosphate procedure. The cells were deprived of serum for 24 h prior to lysis, and were stimulated for 1 h with serum-containing medium to activate Akt kinases. The cells were lysed in 200 μl of 1% Triton buffer (1% Triton X-100, 50 mM Tris-HCl, pH 7.5, 150 mM NaCl, and 1 mM PMSF), the lysates were clarified by centrifugation for 15 min at 13,000 g in a microcentrifuge, and ~100 μg of the supernatants were incubated with 20 μl of protein A-Sepharose coupled to 2 μg of anti-HA or M5 anti-FLAG monoclonal antibodies. Immunoprecipitates were washed three times with lysis buffer and kinase assays were performed on beads as described.

Incubation of GST, GST-LBRNt(62–92), GST-LBRNt(62–92)ΔRS, GST-LBRNt(62–92)S76G, GST-LBRNt(62–92)S78G, GST-LBRNt(62–92)S80A, GST-LBRNt(62–92)S82A and GST-LBRNt(62–92)S84A immobilized on glutathione-Sepharose beads with extracts (~200 μg of total protein) from 293 T cells overexpressing myristoylated HA-Akt1, FLAG-Akt2 and FLAG-SRPK1 was performed in 20 mM Hepes, pH 7.5, 50–150 mM NaCl, 1% Triton X-100, and 0.5 mM phenylmethylsulfonyl fluoride in a total volume of 0.5 ml for 60 min at room temperature. Following incubation, the beads were washed four times (10 min each wash) with incubation buffer and bound HA-Akt1, FLAG-Akt2 and FLAG-SRPK1 were analyzed on 10% SDS-polyacrylamide gels and detected by Western blotting using an anti-HA or an anti-FLAG monoclonal antibody, respectively. An alkaline phosphatase-coupled goat anti mouse was used as secondary antibody, while 5-bromo-4-chloro-3-indolyl phosphate and nitro blue tetrazolium were used as substrates to visualize the immunocomplexes.

### Construction of initial 3D-models

Initial 3D-models of four 10mer LBR peptides bearing phosphosites at residues Ser78, Ser80, Ser82 and Ser84 (LBR-S78: aa 72–81:PSRRSRSRSR, LBR-S80: aa 74–83:RRSRSRSRSR, LBR-S82: aa 76–85:SRSRSRSRSP and LBR-S84: aa 78–87:SRSRSRSPGR, respectively), were constructed in the active site of Akt2 using, as template, the known crystal structure of a GSK3-β 10mer peptide in complex with an activated form (S474D) of human Akt2 kinase domain bound to the non-hydrolysable ATP analog, AMP-PNP (abbreviated as ANP) with attached Mn^2+^ ions (Protein Data Bank (PDB) ID code: 1O6K) [26]. The program Swiss-pdb viewer [27] was used for this purpose. More specifically, coordinates for the backbone atoms of the LBR peptides were extracted from those of the GSK3-peptide in the 1O6K PDB entry, whereas corresponding side-chain atoms were built accordingly using the *mutate* module of the program. Two sets of initial 3D-models were constructed: one set of the four aforementioned Akt2/LBR-peptide complexes and a second set of the same complexes with bound ATP and two Mg^2+^ atoms (hereafter referred to as ATP/MG). For the construction of the latter complexes, the atom names of ANP and manganese atoms of the template crystal structure were changed accordingly to model the ATP and magnesium atoms, respectively. The produced 3D-models of the Akt2/LBR-peptide complexes, as well as the template crystal structure of the Akt2/GSK3-peptide (excluding the coordinates of non-protein atoms) were subsequently used as initial structures for MD simulations both, in the absence (Akt2/peptide complexes; binary) and in the presence of ATP/MG (Akt2/peptide·ATP/MG complexes; ternary). The coordinates of three crystallographic water oxygen atoms coordinated to the Mn^+2^ ions in the 1O6K x-ray structure, were also included in the initial 3D-models of the ternary complexes.

### MD simulations

The MD simulations were carried out in explicit water using periodic dodecahedron boxes of TIP3P water molecules [28] extending 10 Å from protein atoms to solvate the protein systems. Periodic boundaries were applied to minimize edge effects. The systems were neutralized with counter-ions (Na^+^ or Cl^-^ ions, accordingly). The solvated systems were first optimized by the steepest descent energy minimization followed by restrained MD simulations where the protein (and ATP) atoms were harmonically restrained to their initial position with a force constant of 10 kJ/ (mol Å^2^) to allow the solvent to equilibrate. The equilibration step was performed for a total of 200 ps (100 ps under the NVT conditions followed by 100 ps in the NPT ensemble) using the velocity rescaling thermostat [29] and the Parrinello-Rahman barostat [30]. The temperature of the systems was kept at 300 K, with separate coupling of protein and non-protein atoms (including ATP/MG atoms in the ternary complexes). The pressure was kept to 1 bar. The optimization phase was followed by unrestrained MD simulations at 300 K, in the NPT ensemble. To overcome sampling problems, multiple (up to six), independent, 50 ns-long MD simulations were carried out for all eight Akt2/LBR-peptide and Akt2/LBR-peptide·ATP/MG complexes (total of 36 MD trajectories). The replicas were produced using different (random) sets of starting velocities for the atoms. Two additional sets of four and three, independent, 20 and 50 ns-long MD simulations were also performed for the Akt2/GSK3-peptide complex and its modeled ATP/MG-bound form, respectively, for comparison.

All the MD simulations were performed using the GROMACS4 (v. 4.6.3) software package [31] and the AMBER99SB-ILDN force field [32], which produces more reliable MD results, as we have previously shown [4]. The AMBER parameters for ATP with a net charge of -4e [33] (adapted from the Bryce R, AMBER Parameter Database, http://pharmacy.man.ac.uk/amber) were used for the MD simulations in the case of the ternary complexes. The special residue pThr309 of the template crystal structure was replaced by the normal Thr309 and by a Glu residue (as a model for Thr309 phosphorylation) in the MD simulations of the binary and ternary complexes, respectively. The peptide of the template crystal structure (Q466 to I479) bearing the S474D as a model for the required Ser474 phosphorylation [26], was included in all the MD simulations of this study and was treated as a separate protein chain. The particle mesh Ewald method [34] for the treatment of the long-range electrostatic interactions and a 2 fs time step for integration of the potential function, were used in all MD simulations. The LINCS algorithm for covalent bonds [35] and rigid water using the SETTLE algorithm [36], were employed in the MD production runs. A 64-core Dell PowerEdge R815 server was used for all the MD simulations.

### Analysis of the MD simulations

The MD simulations of the Akt2/peptide and Akt2/peptide·ATP/MG complexes studied here were analyzed both separately and on average. Convergence of the MD trajectories was assessed by monitoring the root-mean-square deviation (RMSD) of the backbone atoms from the initial structure along the MD trajectories, using the GROMACS *g_rmsd* module. Cluster analysis used the *g_cluster* module of GROMACS and was carried out for the last 10 and 5 ns of each 50 and 20 ns MD trajectory, respectively, with a backbone RMSD cut-off of 1 Å for two structures to be considered neighbors. Final 3D-models of each simulated Akt2/peptide complex of this study were obtained by averaging the representative structures (structures with the smallest average RMSD from all other structures of a cluster; referred to as MD models) of their corresponding sets of MD simulations, followed by optimization by the steepest descent energy minimization with flexible water. Structure averaging, root-mean-square atomic fluctuation (RMSF) calculations and estimation of corresponding temperature-factors (B-factor) were carried out using the *g_rmsf* analysis tool of GROMACS. The RMSFs and atomic B-factors were used as a measure of the conformational flexibility of the simulated systems. RMSF values of the backbone atoms referenced to the average structure were calculated over the last 10 ns and 5 ns of each 50 ns and 20 ns MD trajectory, respectively and were subsequently averaged within each set of multiple MD trajectories. Atomic B-factors were estimated from atomic fluctuations of the multiple MD models relative to their average structure within the corresponding MD model sets of each simulated system, whereas the crystallographic B-factors were used in the case of the template crystal structure. The VMD program [37] was employed for visualization of the trajectories, whereas molecular model illustrations were rendered using VMD and PyMOL (The PyMOL Molecular Graphics System, Version 1.7.0.0. Schrödinger, LLC).

## Results and Discussion

### *In vitro* phosphorylation of LBRNt(62–92) by SRPK1 and activated Akt

**Fig 1:**
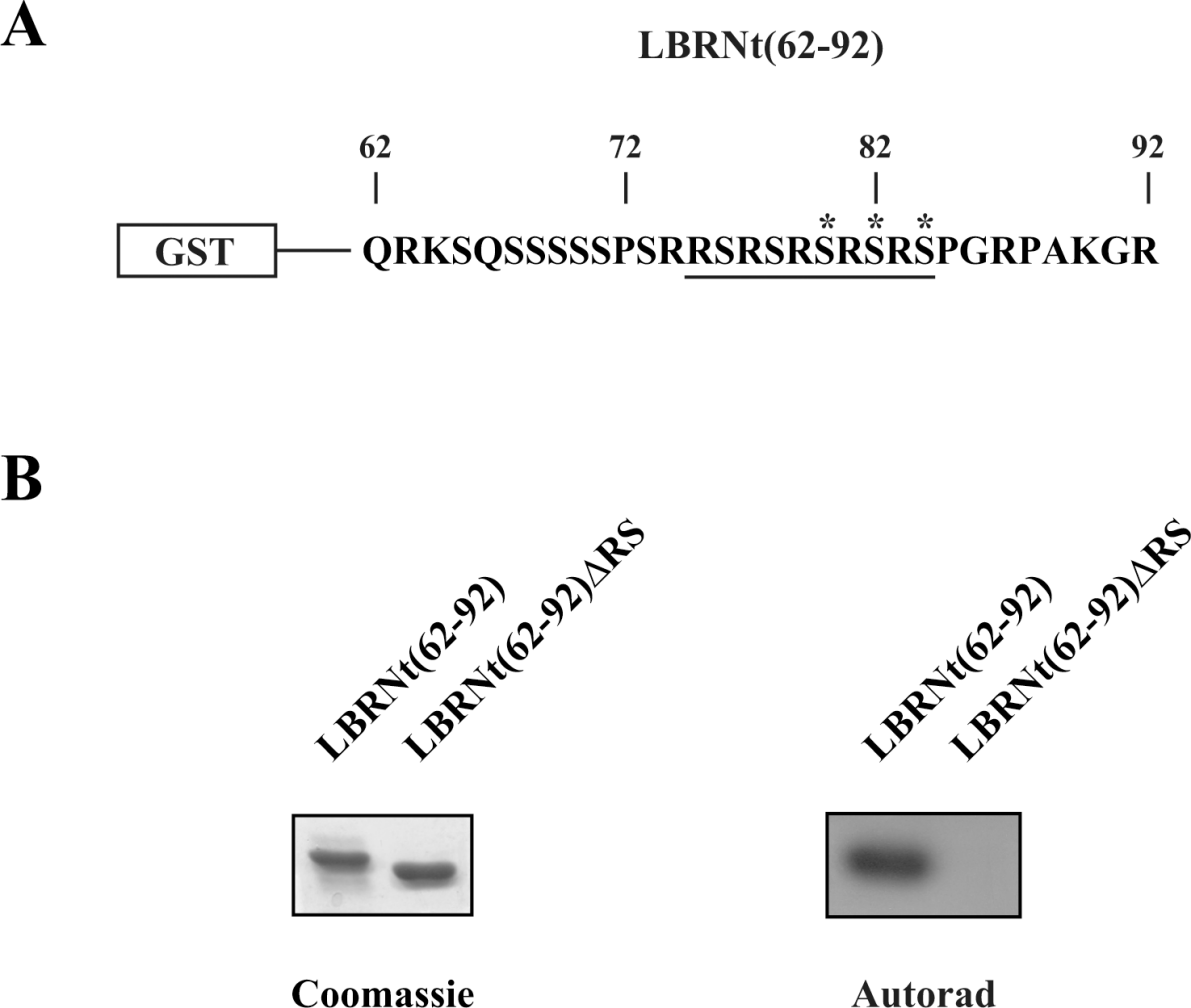
Phosphorylation of LBRNt(62–92) and LBRNt(62–92)ΔRS by Akt1. (A) Amino acid sequence of LBRNt(62–92). The RS domain is underlined and the putative Akt sites (Ser80, Ser82 and Ser84) are marked with an asterisk. (B) Phosphorylation of GST-LBRNt(62–92) and GST-LBRNt(62–92)ΔRS by 0.07 **μ**M Akt1. The samples were analyzed by SDS-PAGE on 12% gels, stained with Coomassie Blue and autoradiographed.

Both SRPK and Akt kinases have been implicated in the phosphorylation of serine residues within RS domains. Yet, while there is no doubt that SRPKs act directly on RS domains, there are several concerns on the direct phosphorylation of RS domain-containing proteins by activated Akt. In this respect, LBR, a well-studied substrate of SRPK1, was used first to analyze the ability of activated Akt to target directly RS dipeptides and then to compare the specificity of the two kinase families. The RS domain of turkey LBR (Fig 1A, sequence underlined) is composed of five consecutive RS repeats which are all targeted by SRPK1 [21, 22]. Within this domain there are three putative Akt sites (Ser80, Ser82 and Ser84, marked with an asterisk in Fig 1A) that conform to the minimum Akt consensus RXRXXS/T. To drive the phosphorylation by activated Akt exclusively to the serines of the RS motif we expressed in *E*. *coli*, as fusion protein with GST, a fragment of the N-terminal domain of turkey LBR that contains the RS dipeptides but lacks any other putative Akt sites (construct termed GST-LBRNt(62–92), Fig 1A). Accordingly, GST-LBRNt(62–92) was phosphorylated by recombinant active Akt1 (Upstate Biotechnology), whereas a similar recombinant protein missing the RS dipeptides (construct termed GST-LBRNt(62–92)ΔRS) was not (Fig 1B).

The apparent Km value displayed by Akt1 for the phosphorylation of GST-LBRNt(62–92) was 0.465 μM and was significantly lower than the previously reported value (5 μM) for the authentic Akt substrate, H2B [38].

We then proceeded to analyze whether the phosphorylation of GST-LBRNt(62–92) by Akt1 was direct. One of the major clues contradicting the assumption that RS domains are directly phosphorylated by Akt kinases is the substrate competition assay performed by Zhou et al. [14]. According to the data reported, titration of the classical Akt substrate GSK3β suppressed the Akt kinase activity on another well-characterized Akt substrate, H2B, but had no effect on a typical RS domain-containing protein, SRSF1. Conversely, a synthetic SRPK substrate containing 16 Ser/Arg repeats (SR16), that was able to suppress the Akt kinase activity toward SRSF1, not only was unable to suppress the kinase activity towards GSK3β but significantly enhanced it. The outcome of these experiments led the authors to the suggestion that both Akt and SR kinase activities are present in even highly purified constitutively active Akt from a commercial source [14].

**Fig 2:**
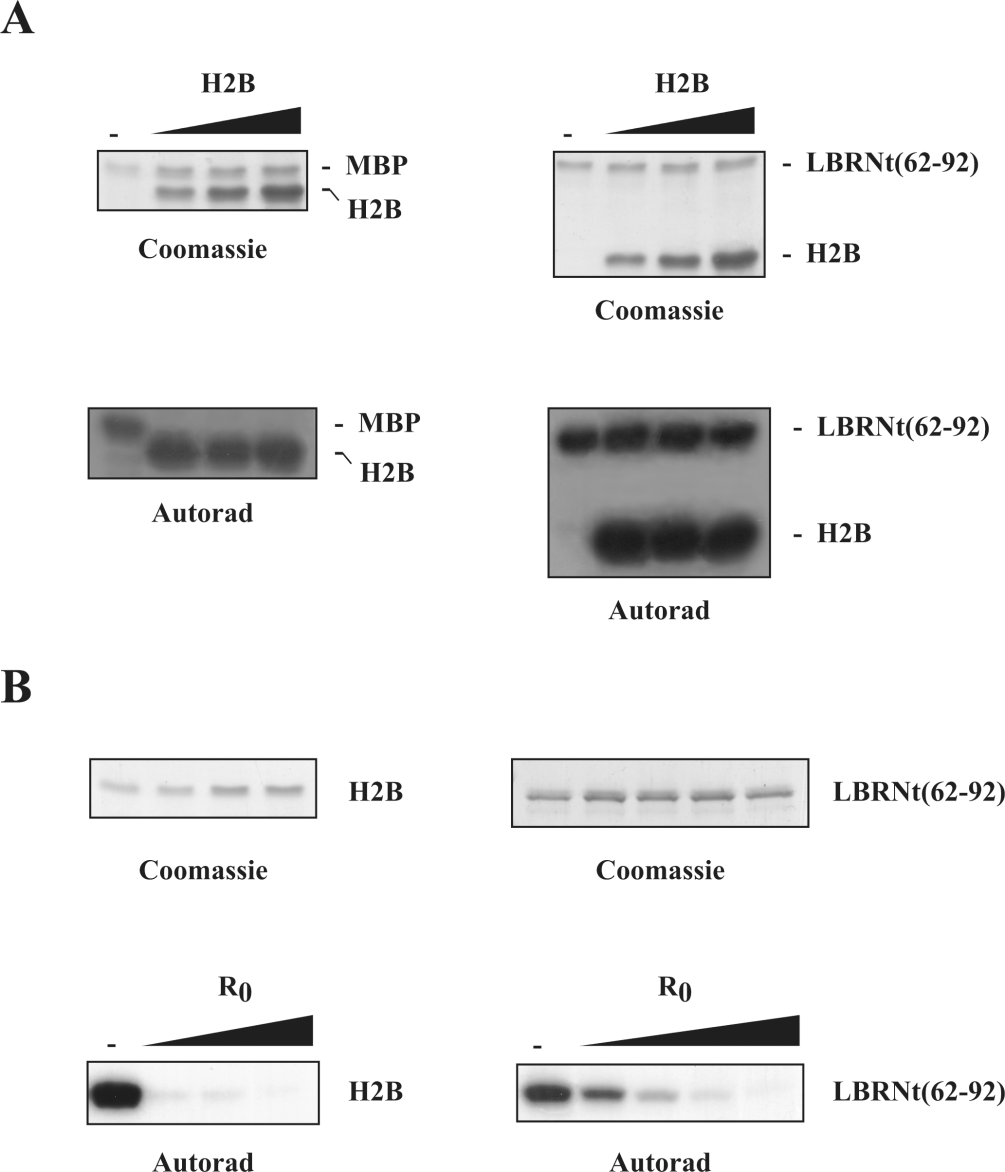
Substrate competition assays. (A) Phosphorylation of MBP (1.1 μM) and GST-LBRNt(62–92) (0.7 μM) by Akt1 (0.07 μM) in the presence of increasing concentrations of H2B (2.8, 5.6 and 5.6 μM). Upper panel, Coomassie blue staining; lower panel autoradiography. (B) Phosphorylation of H2B (1.4 μM) and GST-LBRNt(62–92) (0.7 μM) by Akt1 (0.07 μM) in the presence of increasing concentrations of R0 peptide (125, 250, 375 and 500 μM). Upper panel, Coomassie blue staining; lower panel autoradiography.

To delineate this issue we repeated the substrate competition assay using recombinant active Akt1, two classical Akt substrates, MBP and H2B, and two SRPK substrates, GST-LBRNt(62–92) and a synthetic peptide derived from the amino-terminal region of turkey LBR that contains the entire RS sequence (R0). We found that, while increasing concentrations of H2B were able to fully suppress MBP phosphorylation they had no effect towards GST-LBRNt(62–92) (Fig 2A). On the contrary, more elevated concentrations of the R0 peptide were required to fully inhibit the phosphorylation GST-LBRNt(62–92) than the phosphorylation of H2B (Fig 2B). These data strengthen the standpoint that GST-LBRNt(62–92) functions not only as a direct substrate of Akt kinases but it is even phosphorylated to a higher extent than H2B because it contains more targeted sites.

### Akt phosphorylates distinct sites than SRPK1 within the RS domain of LBR

According to Scansite Motif Scanner (http://scansite.mit.edu/motifscan_seq.phtml), a motif-profile scoring algorithm generated by Yaffe and Cantley, which takes into consideration not only the phosphorylation motif but also the positive or negative influence of the flanking residues [39], Ser80, Ser82 and Ser84 are all considered as high-stringency hits since they fall within the top 0.2% of all the potential Akt-phosphorylation sites contained in vertebrate Swiss-Prot proteins (the score percentages for the three serines are 0.073%, 0.104% and 0.069%, respectively).

**Fig 3:**
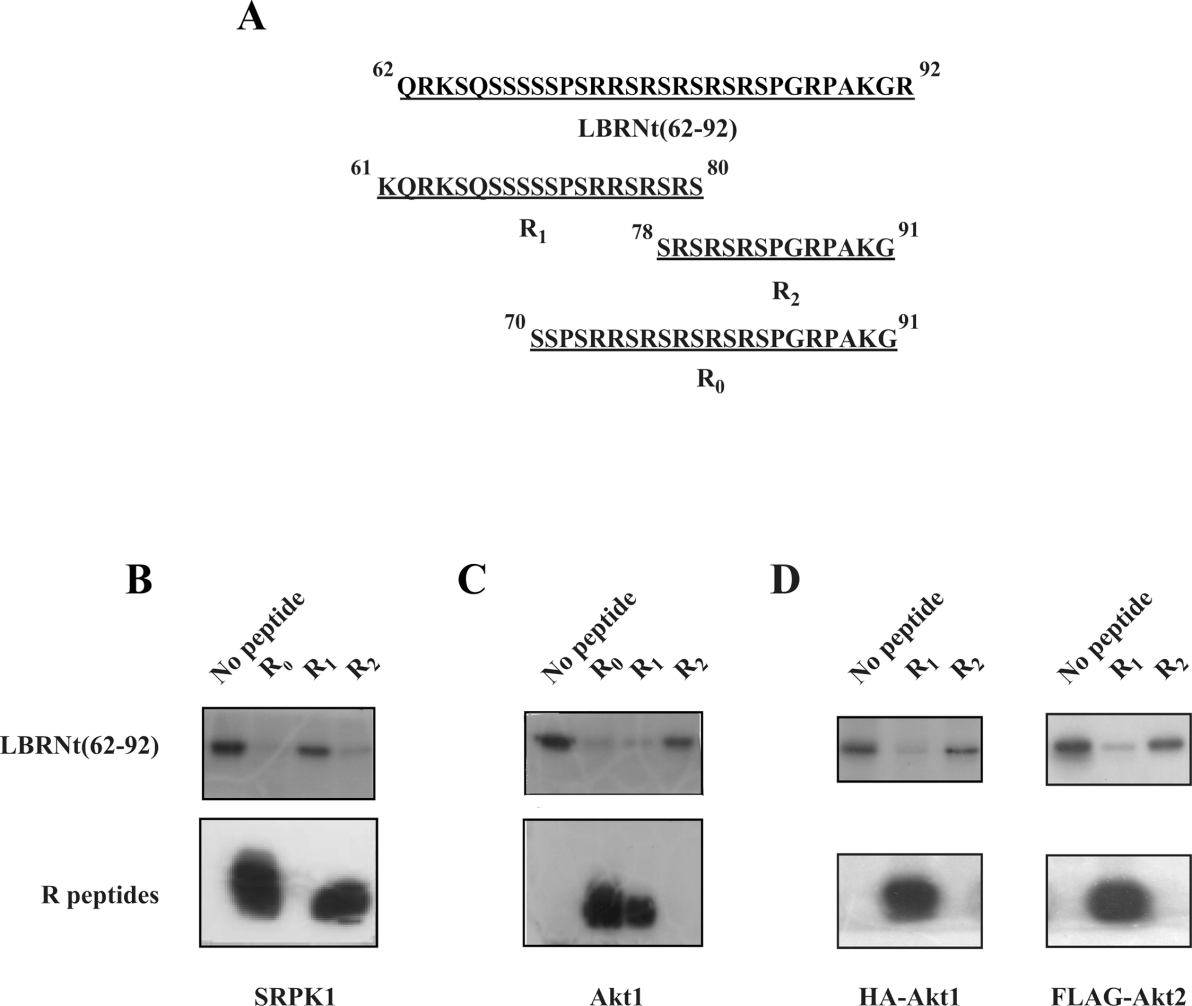
Phosphorylation of LBRNt(62–92) by GST-SRPK1 and Akt1 in the presence of different synthetic peptides. (A) Amino acid sequences of the peptides used. The relative position of the peptides in LBRNt(62–92) is schematically indicated. 1.95 μM GST-LBRNt(62–92) were incubated with 0.19 μM GST-SRPK1 (B) or 0.07 μM recombinant Akt1 (C) or immunoprecipitated myristoylated HA-Akt1 and FLAG-Akt2 (D) in the presence of 500 μM of each peptide and 25 μM [γ- ^32^P]ATP as described under “Materials and Methods”. Samples were subsequently analyzed by SDS-PAGE on a 15% gel and autoradiographed. In (B), (C) and (D) phosphorylation of GST-LBRNt(62–92) is shown in the upper panel and phosphorylation of the peptides is shown in the lower panel.

In a first attempt to probe into the specificity of activated Akt and to compare it with the specificity and mode of action of SRPK1 we used three subfragment peptides of LBRNt(62–92) that contain varying number of RS dipeptides (Fig 3A) and tested their ability to compete with GST-LBRNt(62–92) phosphorylation by SRPK1 and Akt. As previously shown [22], two of the peptides tested: R0, which contained the entire RS sequence plus a downstream sequence and R2, which contained the three C-terminal RS dipeptides and the same downstream sequence, could be phosphorylated by GST-SRPK1 and inhibited almost completely the phosphorylation of GST-LBRNt(62–92) (Fig 3B). The third peptide, R1, which contained the three N-terminal RS dipeptides but no downstream flanking sequence could not serve as substrate and affected GST-LBRNt(62–92) phosphorylation marginally (Fig 3B). Similarly to SRPK1, R0 peptide could also be efficiently phosphorylated by Akt1 and inhibited the phosphorylation of GST-LBRNt(62–92). Yet, conversely to SRPK1, R1 could be phosphorylated by Akt1 and was able to inhibit the phosphorylation of GST-LBRNt(62–92), whereas R2 could not function as a substrate of Akt1 (Fig 3C). Moreover, since in most of the previous studies immunopurified Akt that may contain traces of other co-precipitating kinases (including SRPK1), was used as a kinase source to phosphorylate various SR proteins [16, 19, 20], we repeated the phosphorylation assays using immunoprecipitated myristoylated HA-Akt1 and FLAG-Akt2, in the presence of the inhibitory peptides R1 and R2 that clearly discriminate between Akt and SRPK activity. Similarly to commercially purchased recombinant Akt, R1 could be phosphorylated by immunoprecipitated kinases and was able to inhibit the phosphorylation of GST-LBRNt(62–92), whereas R2 was inactive (Fig 3D). Thus, it seems that while the C-terminal region of the LBR RS domain constitutes a recognition platform for SRPK1 and is required for phosphorylation [4, 22], Akt kinases require also upstream sequence.

To determine more specifically the serine residues of LBR that are phosphorylated by Akt kinases, we mutated each individual serine of the RS domain to glycine or alanine. More specifically, Ser76, Ser78, Ser80, Ser82 and Ser84 were mutated to Gly (LBRNt(62–92)S76G), Gly (LBRNt(62–92)S78G), Ala (LBRNt(62–92)S80A), Ala (LBRNt(62–92)S82A) and Ala (LBRNt(62–92)S84A), respectively. Previous bioinformatics analysis of the RS domain of turkey LBR predicted that this protein fragment is totally disordered [40]. Subsequent *ab-initio* folding MD experiments of short peptides containing four or five consecutive RS dipeptides predicted that the conformation of such peptides, in isolation, is indeed flexible than well structured [4]. Therefore, replacement of serine residues of the LBR RS-domain by alanine or glycine (although with different helix forming propensities) is highly unlike to further unfold or misfold this domain.

**Fig 4:**
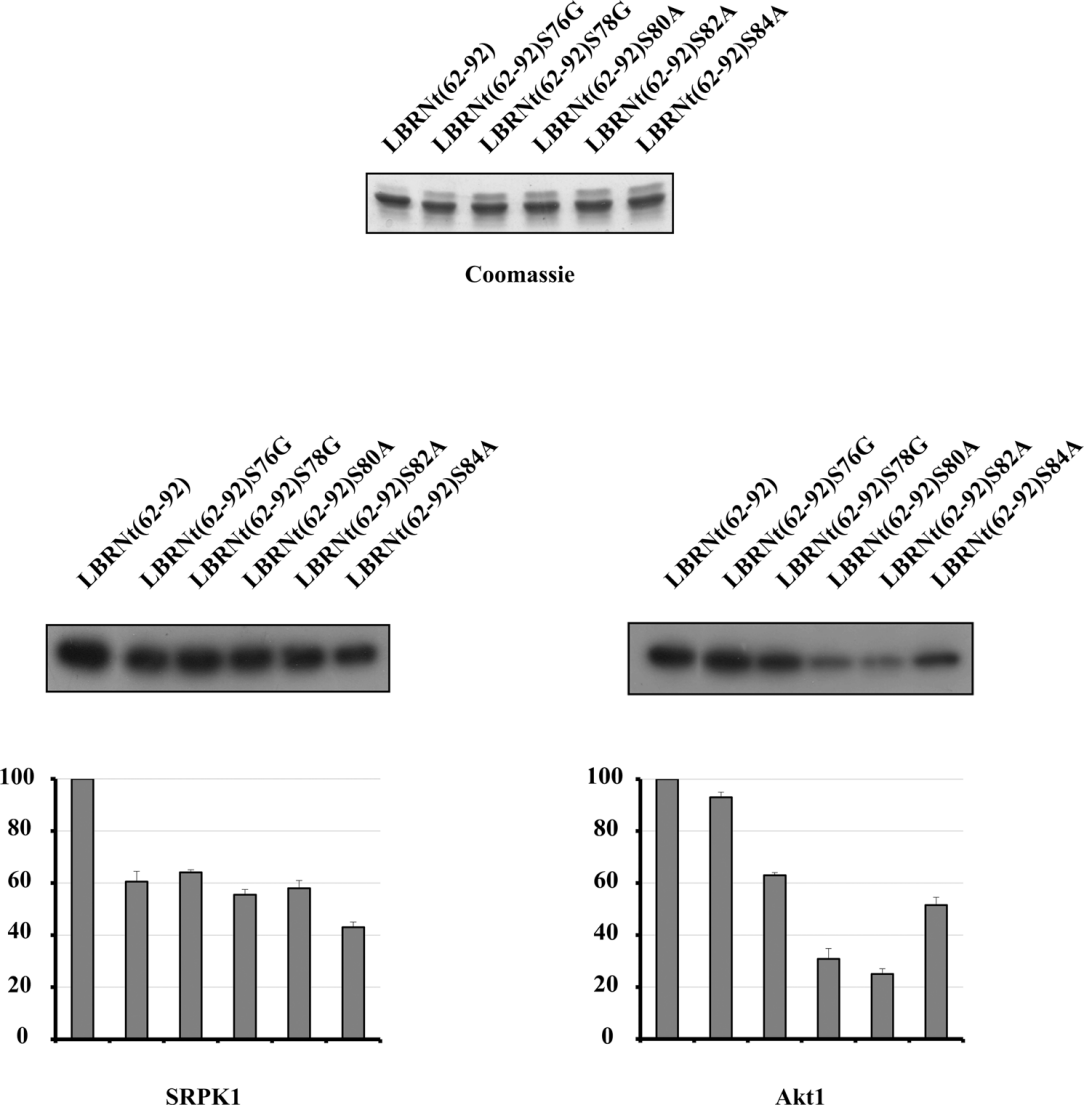
Determination of the sites phosphorylated by SRPK1 and Akt1. Phosphorylation of GST-LBRNt(62–92), GST-LBRNt(62–92)S76G, GST-LBRNt(62–92)S78G, GST-LBRNt(62–92)S80A, GST-LBRNt(62–92)S82A και GST-LBRNt(62–92)S84A by 0.19 μM GST-SRPK1 (left panel) and 0.07 μM Akt1 (right panel). Only the relevant part of the autorad corresponding to the phosphorylated recombinant proteins is shown. Enzyme activity is expressed as a percent of the activity obtained with GST-LBRNt(62–92) which was set to 100 percent. Data represent the means ± SE of three independent experiments. On top of the figure we show a Coomassie Blue staining of the recombinant proteins (1.95 μM of each) used in the phosphorylation assays.

All recombinant proteins were appropriately expressed, purified, and used as substrates for *in vitro* phosphorylation assays with SRPK1 and Akt1. The rate of phosphorylation of the single mutants by SRPK1 was similar and at about 50% of the wild type, except LBRNt(62–92)S84A which was phosphorylated to a slightly lesser extent (Fig 4, left panel). Mutation of either of the first two serines (Ser80 and Ser82) that conform to the consensus recognized by Akt, to alanine, severally inhibited phosphorylation by Akt1, while mutation of the third serine (Ser84) to alanine was less critical (Fig 4, right panel). Similar results were obtained when the above experiments were repeated using recombinant active Akt2 (Life Technologies) instead of Akt1 or immunoprecipitated myristoylated HA-Akt1 and FLAG-Akt2 (data not shown), further confirming that Akt1 and Akt2 act directly on the LBR RS domain and exhibit the same specificity.

### Binding of Akt and SRPK1 to the RS domain of LBR

To test whether the reduced phosphorylation of Ser84 as compared to Ser80 and Ser82 is due to reduced binding to activated Akt, we performed pull-down experiments. To this purpose, GST, GST-LBRNt(62–92) and the recombinant mutant proteins immobilized on glutathione-Sepharose beads were incubated with extracts from 293 T cells overexpressing myristoylated HA-Akt1. To our surprise we did not observe any binding even at low concentrations of NaCl (50 mM). We also failed to observe any binding when we used extracts from 293 T cells overexpressing myristoylated FLAG-Akt2, suggesting that the inability of activated Akt to associate with GST-LBRNt(62–92) was not an Akt isoform-specific effect. When Mg^2+^ (2 mM) was included in the binding buffer to achieve binding, a considerable amount of both HA-Akt1 and FLAG-Akt2 was found to associate with glutathione-sepharose beads alone. Even though, theoretically, Mg^2+^ may promote an interaction of Akt with the beads we assumed that, in the presence of Mg^2+^, Akt had the tendency to precipitate. Conversely, FLAG-SRPK1 was able to associate with GST-LBRNt(62–92) and with the recombinant mutant proteins except GST-LBRNt(62–92)ΔRS as previously shown [4, 41].

### Dynamics of LBR substrate peptides in the active site of Akt2: Insights from MD simulations

**Fig 5:**
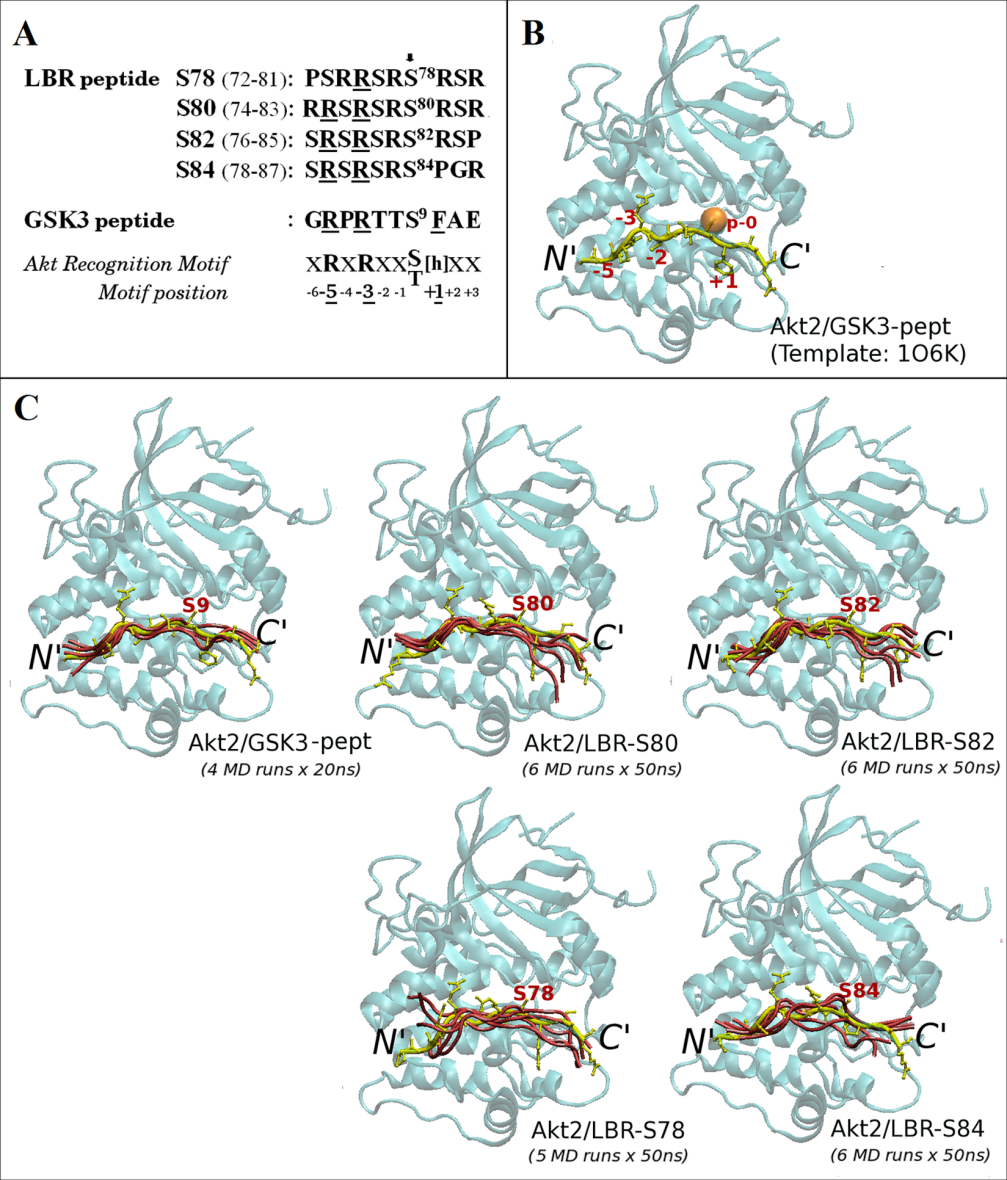
3D-modeling of LBR peptides in complex with Akt2. (A) Sequence alignment of overlapping LBR peptides used for 3D-modeling of Akt2/LBR-peptide complexes and of the GSK3-β peptide. The consensus sequence of the Akt recognition motif is also shown. h stands for large hydrophobic aminoacids. Potential phosphoacceptor serine residues are indicated by an arrow. (B) The known crystal structure of the GSK3-β peptide (in tube) bound to the kinase domain of human Akt2 (in ribbon), used as the modeling template (1O6K) [26]. Substrate peptide positions are numbered according to the Akt consensus shown in A. The phosphoacceptor site is indicated by an orange sphere. (C) Results of multiple, independent MD simulations (S1 Table) of the LBR peptides shown in A and of the GSK3-β peptide in complex with human Akt2 (as in B). Only the multiple MD models (and the corresponding initial conformations; in yellow) of the bound peptides are shown for clarity. The phosphoacceptor serines are labeled. Molecular model illustrations were rendered using VMD [37].

It has been reported that the presence of a large hydrophobic residue immediately C-terminal to the phosphorylated serine is critical for efficient phosphorylation by Akt kinases [38]. Yet, Ser80 and Ser82 function as efficient phosphoacceptor sites (Fig 4, right panel) despite the absence of a hydrophobic residue at this position (Fig 5A). On the other hand, Ser84, which is followed by a proline residue (Fig 5A), is a less efficient Akt1 site (Fig 4, right panel) in agreement with data showing that, the presence of a proline at susbstrate position +1 prevents phosphorylation by Akt [38]. In contrast, and despite the absence of the Akt consensus arginine at motif position -5 (Fig 5A), Ser78 represents a weak but yet a recognizable site by Akt, as indicated by the lower phosphorylation of LBRNt(62–92)S78G (Fig 4, right panel). One possible explanation is that PSRRSRSRSR (Ser78 is the underlined serine) functions as a weak Akt recognition motif. To address these issues, we sought to understand the structural basis of our findings so far.

Towards this end, the known crystal structure (Fig 5B) of a GSK3-β peptide in complex with an activated form of Akt2 (S474D) bound to ANP/MN (PDB ID code: 1O6K) [26] was used as modeling template, as described under “Materials & Methods”. Our approach was based on the assumption that each individual Akt site of the RS domain functions as the unique site of GSK3-β. In this respect, we first produced 3D-models of four, overlapping, 10mer LBR peptides corresponding to potential Akt recognition sequences (Fig 5A and S1 Fig), into the active site of Akt2 (Fig 5C; peptides in yellow).

#### MD simulations of binary Akt2/LBR-peptide complexes

The initial 3D-models of each of the four Akt2/LBR-peptide complexes where subsequently subjected to 50 ns-long MD simulations in explicit water and in the absence of ATP/MG atoms, as described under “Materials & Methods”. Because of the stochastic nature of protein MD simulations and to obtain more reliable MD results (to reduce sampling problems), multiple (up to six), independent MD simulations for each Akt2/LBR-peptide binary complex were carried out. An additional set of four, independent, 20 ns-long MD simulations was also carried out for the Akt2/GSK3-peptide binary complex, for comparison. Convergence of the MD trajectories was assessed by monitoring the RMSD (see “Materials & Methods”) of the backbone atoms of all five binary Akt2-peptide complexes relative to their corresponding initial 3D-models, along the multiple MD trajectories. The simulated systems had reached equilibrium (at backbone RMSD values ≤ 2 Å) and remained stable at least during the last 10 ns and 5 ns of each 50 ns and 20 ns trajectory, respectively (S2 Fig, left panel) and this simulation time range was subsequently used for further analysis of the MD trajectories. Cluster analysis (see “Materials & Methods”) yielded only one cluster of structures with backbone RMSD values of less than 1Å (~100% of structures), for all the Akt2/peptide MD trajectories. Peptide conformations extracted from the representative structure of each MD cluster (structure with the most neighbors; details in S1 Table) of the binary complexes, are shown in Fig 5C (pink tubes). It should be noted here, that since the kinase domain of human Akt1 shares 91% sequence similarity and 81% identity with that of human Akt2 (or 95% similarity and 88% identity for the Akt2 region determined in the 1O6K crystal structure; S3 Fig), and both kinases were shown to exhibit the same specificity on the LBR RS domain, use of Akt1 sequence unlikely changes the MD results presented hereafter.

**Fig 6:**
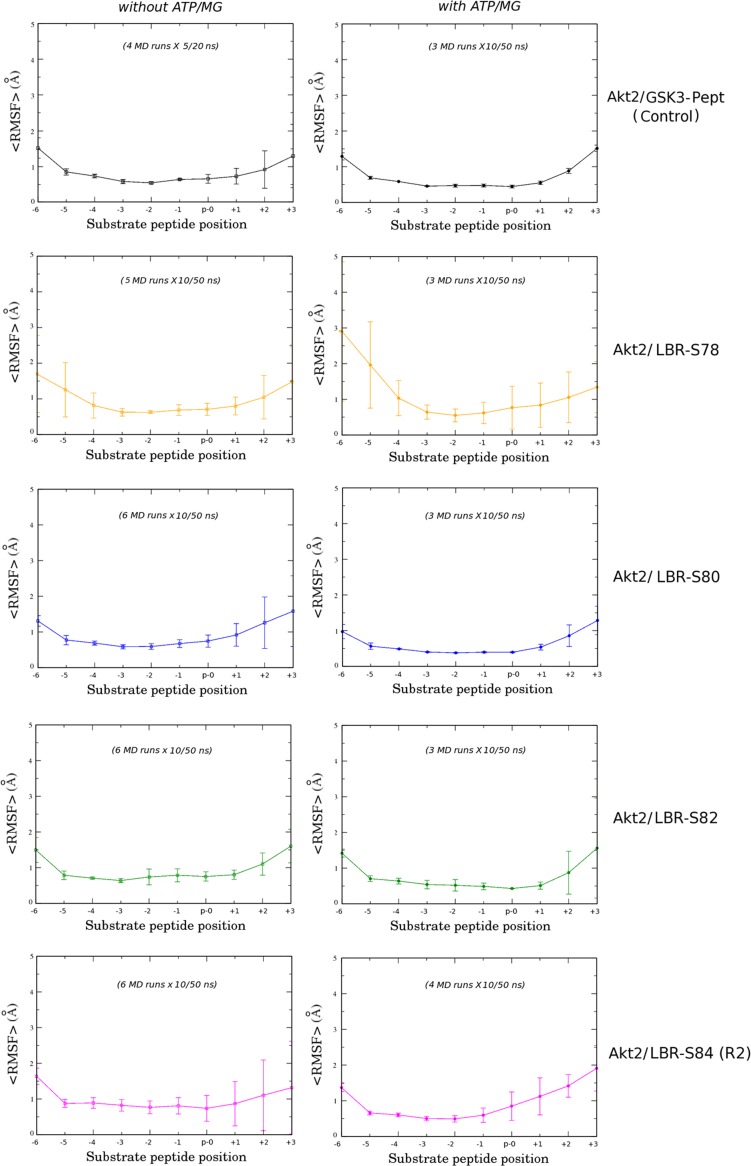
Backbone fluctuations of the simulated GSK3-β and LBR peptides bound to Akt2. Average RMSF values (see “Materials & Methods”) obtained from the corresponding sets of MD simulations of the (left) binary and (right) ternary complexes simulated in this study. Bar lines represent corresponding standard deviations from the mean value within each MD simulation set. Substrate peptide positions are numbered according to the Akt consensus motif (Fig 5A).

As shown in Fig 5C and also reflected by the similar root-mean-square fluctuations (RMSF) of their backbone atoms within the corresponding sets of MD simulations (Fig 6, left panel), the conformational fluctuations of the LBR-S80 and LBR-S82 peptides into the active site of Akt2 resemble those of the GSK3-β peptide (Fig 5C, top). On the other hand, peptides LBR-S78 and LBR-S84 exhibited a much more dynamic behavior throughout their length, including the region around their phosphosites (Fig 5C, bottom and Fig 6, left panel). In addition, from our MD results so far, it appears that the region C-terminal the phosphosite of even the optimal LBR-S82 and LBR-S80 peptides (including that of the GSK3 substrate peptide) exhibits higher atomic fluctuations than the rest of the peptides (Fig 5C, bottom and Fig 6, left panel). This observation, in conjunction with the fact that the Akt2/GSK3-peptide complex was crystallized in the presence of the non-hydrolysable analog of ATP, ANP, implies that stabilization of the LBR substrate-peptides (and even of the GSK3 peptide) into the active site of Akt requires the presence of ATP, as also observed in the case of Akt peptidomimetic inhibitors [42].

#### MD simulations of ternary Akt2/peptide complexes with ATP

Additional multiple (up to four) MD simulations were carried out for each one of the five Akt2/peptide complexes, in the presence of ATP/MG atoms (ternary complexes; see “Materials & Methods”). All simulated systems reached equilibrium (at backbone RMSD values < 2 Å) and remained stable at least during the last 10 ns of each 50 ns trajectory (S2 Fig, right panel). As reflected by the much lower mean RMSF values of the backbone atoms (Fig 6, right panel), the entire region of the GSK3-, LBR-S80 and LBR-S82 peptides comprising the Akt recognition consensus sequence (substrate positions -5 to +1), was indeed stabilized into the active site of Akt2 in the presence of ATP/MG atoms. On the contrary, the LBR-S78 and LBR-S84 peptides, and most importantly, the region around their phosphosites, remained highly dynamic also in the corresponding ternary complexes (Fig 6). These observations suggest that the former LBR peptides can act, at least as optimal Akt substrates as GSK3, whereas peptides LBR-S78 and LBR-S84 are less efficient, in perfect agreement with the phosphorylation results shown in Fig 4 (right panel). Taken together, our results so far suggest that both the region N-terminal the phosphosite as well as the adjacent to the phosphoserine, site +1, are important for phosphorylation of LBR by Akt.

#### Atomic details of the Akt2/LBR-peptide interactions in the simulated ternary complexes

To unravel the atomic details of the specific interactions of the bound LBR peptides within the active site of Akt, the multiple models derived from the corresponding sets of MD simulations (details given in S1 and S2 Tables) were averaged and optimized by energy minimization to obtain one final model of each simulated Akt2/substrate-peptide complex.

**Fig 7:**
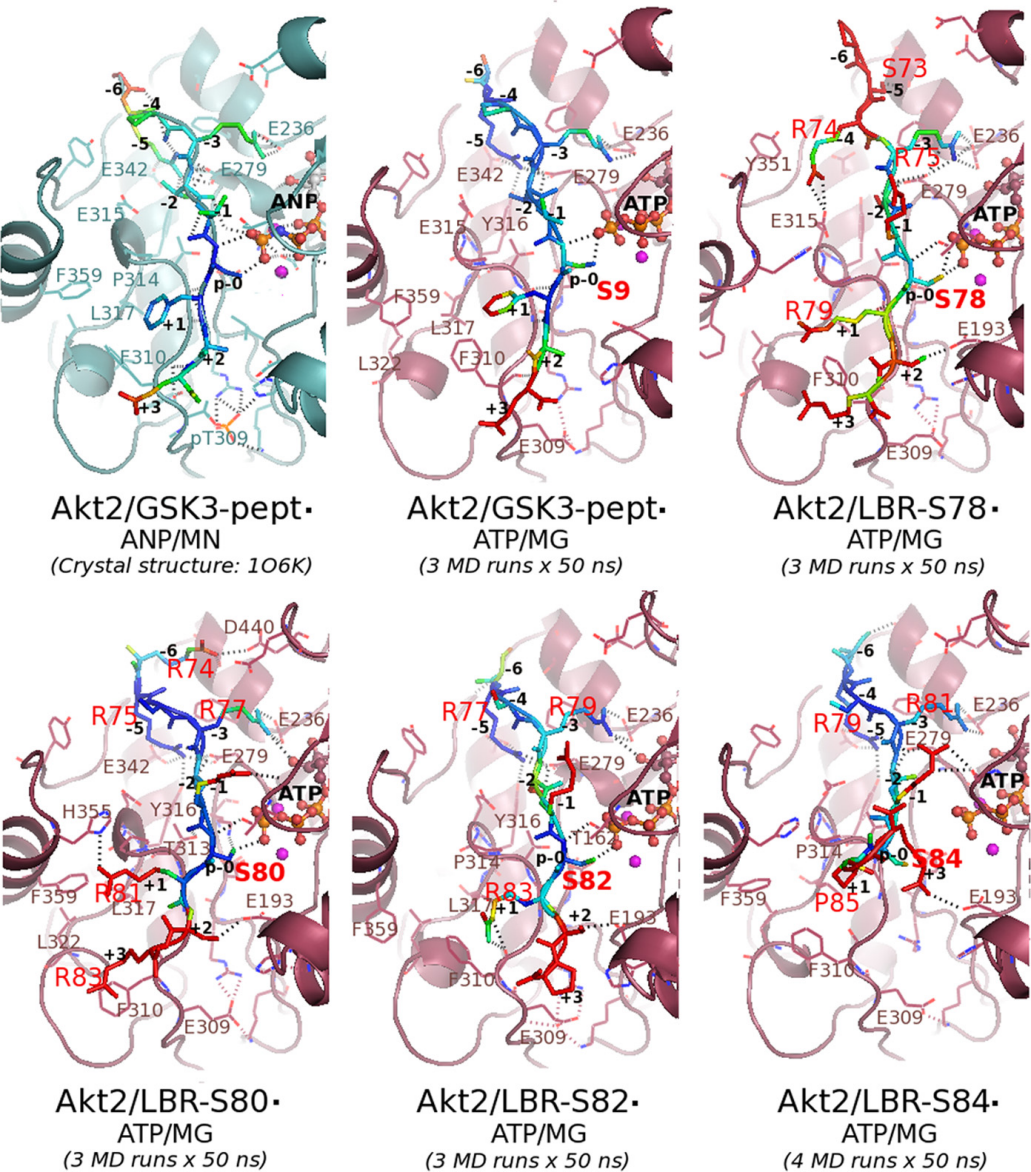
Atomic details of the interactions of LBR peptides with Akt2 in the simulated ternary complexes. Details of the interactions of the GSK3-β peptide with Akt2 in the template crystal structure and in the corresponding final 3D-model produced in this study are also shown. The bound peptides are depicted as stick models and are colored according to their atomic B-factors (see “Materials & Methods”): from blue to red for low to high B-factor values, respectively. Critical residues are labeled. Peptide positions are numbered according to the Akt consensus motif. The ANP and ATP molecules are depicted as balls-and-sticks. Magnesium (manganese in the crystal structure) atoms are shown as spheres, in magenta. H-bond interactions are depicted as broken lines. This figure was rendered using PyMOL.

Details of the final 3D-models (energy minimized average MD models) of all the simulated ternary Akt2/peptide·ATP/MG complexes are shown in Fig 7.

As expected for a catalytically competent conformation, the hydroxyl group of serine residues at p-0 positions was found to be located within a hydrogen-bonding (H-bond) distance from the γ-phosphate group of ATP in the case of the GSK3, LBR-S80 and LBR-S82 peptides, but not in the case of the LBR-S84 peptide (Fig 7 and Table 1), in line with the phosphorylation results. Surprisingly however, an equally optimal positioning of the p-0 site was observed in the case of the LBR-S78 peptide (Fig 7 and Table 1) (see below).

**Table 1:** Distance between phosphosites and ATP γ-phosphate in the ternary complexes.

System	S_p0_-O^γ^…P^γ^-ATP Distance(Å)	B-factor (S_p0_-O^γ^) (Å^2^)
Akt2/GSK3-pept · ANP/MN (crystal structure: 1O6K)	3.57	14.8
Akt2/GSK3-pept · ATP/MG	3.35	13.6
Akt2/LBR-S78 · ATP/MG	3.65	65.8
Akt2/LBR-S80 · ATP/MG	3.44	36.5
Akt2/LBR-S82 · ATP/MG	3.59	44.9
Akt2/LBR-S84 · ATP/MG	6.63	67.9

Distance between the hydroxyl oxygen of the corresponding serine residues at p-0 peptide positions (S_p0_-O^γ^) and the γ-phosphorus atom of ATP (P^γ^-ATP), and calculated B-factor values (see “Materials & Methods”) of the S_p0_-O^γ^ atoms, in the final 3D-models of the ATP/MG-bound Akt2/peptide complexes simulated in this study (shown in Fig 7). The equivalent, S_9_-O^γ^…P^γ^-ANP distance and crystallographic B-factor in the template crystal structure, are also given for comparison.

#### Substrate-peptide positions N-terminal the phosphosite

As revealed by comparison of the atomic details of the produced final 3D-models of the simulated ternary complexes with those of the template crystal structure, the side chain of the arginine residues corresponding to motif positions -3 (R75, R77, R79 and R81 for the LBR peptides) preserve the salt-bridge interaction with Glu236, a key acidic residue of the Akt2 active site, in all cases (compare interactions of -3 Args in Fig 7). This interaction was also observed in the case of the simulated binary complexes (data not shown). This observation is in perfect agreement with the compulsory Arg specificity at position -3 of Akt substrates [43].

Interestingly, in the case of the LBR-S78 peptide the equivalent arginine (R75) appeared to be shared between Glu236 and Glu279 (Fig 7; Akt2/LBR-S78), an acidic residue that is normally part of the P-5 subsite of the substrate peptide-binding site of Akt2 [26] (see also Fig 7; Akt2/GSK3). Indeed, the side chain of Glu279 formed salt-bridge interactions with the side chain of the equivalent arginine at -5 in all LBR peptides with the exception of LBR-S78, which lacks an arginine at this position (Fig 7). Instead, in the case of the latter peptide the side chain of the preceding arginine (R74, at motif position -4), appeared to be placed within a H-bond distance from another important acidic residue of Akt2, Glu315 (Fig 7; Akt2/LBR-S78), which is normally part of the P-2 site in serine/threonine kinases [44] (specificity determinant 16). This interaction (also observed in the final 3D-model of the Akt2/LBR-S78 binary complex; data not shown) may contribute to the ability of Ser78 to act as a weak Akt substrate despite the lack of the consensus arginine at -5. Interestingly enough, and as already mentioned, in the case of the Akt2/LBR-S78·ATP/MG final 3D-model, the average distance of the side chain of Ser78 from the ATP γ-phosphate was estimated to be less than 4 Å, a positioning adequate for phosphorylation and comparable to that in the case of the ATP/MG-bound Akt2/GSK3-peptide, Akt2/LBR-S80 and Akt2/LBR-S82 complexes (Fig 7 and Table 1). However, as suggested by the estimated high B-factor (high mobility) of its hydroxyl oxygen (Table 1) and by high backbone RMSF values (Fig 6, right panel), this positioning of Ser78 is probably transient, in line with the idea that the LBR-Ser78 acts as a weak Akt substrate. In general, a high negative correlation (Pearson correlation coefficient = -0.88) was observed between phosphorylation efficiency of LBR sites and the mobility of their hydroxyl oxygens in the corresponding simulated ternary Akt/LBR-peptide complexes, i.e. the higher the B-factor of the hydroxyl oxygen of the potential phosphoacceptor site the lower its phosphorylation efficiency (see S4 Fig).

Taken together our findings so far suggest that residues, especially arginines, at substrate positions flanking the -5 position, may mimic or even reinforce the Akt recognition sequence. Indeed, an extra arginine at position -6 of the LBR-S80 peptide (R74, Fig 5A), was found within a H-bond distance from Asp440 in both the binary (data not shown) and the ternary complexes (Fig 7; Akt2/LBR-S80). Asp440 is another important acidic residue, part of the P-3 subsite in other serine/threonine kinases, including Akt2 in the case of its GSK3 substrate [26] (see also Fig 7). In further support to this notion, arginine at position -7 was also found to contribute to recognition of substrates by Akt [43].

#### Substrate-peptide position +1

As already mentioned, substrate-peptide position +1 is also crucial for recognition by Akt kinases and this position is usually occupied by large hydrophobic residues [38], a character dictated by the hydrophobic nature of the P+1 pocket of the substrate-peptide binding site of this kinase [26]. Indeed, in the case of the GSK3-β peptide, position +1 is occupied by a phenylalanine, the bulky side chain of which, is well accommodated into the P+1 hydrophobic pocket of Akt2 formed by large hydrophobic residues, including Phe310, Leu317 and a proline residue, P314 [26] (corresponding to site +1 specificity determinants: 13, 18 and 15, respectively, in Ser/Thr kinases [44]). This pocket is further enlarged in Akt kinases by the presence of an additional phenylalanine residue (Phe359 in Akt2)[26] (see also Fig 7).

In the case of the LBR peptides modeled here, with the exception of the LBR-S84 case, position +1 is occupied by an arginine (Fig 5A), which normally does not comply with the consensus hydrophobic character of this position for Akt substrates. It is now well established however, that the guanidinium group of arginine residues is able to form among others, cation-π interactions with the side chains of aromatic residues and as such this type of interactions is now recognized as an important contributor to protein-protein interfaces [45]. Indeed, in the case of the ATP/MG bound Akt2/LBR-S78, Akt2/LBR-S80 and Akt2/LBR-S82 complexes, the +1 Arg (R79, R81 and R83, respectively) appeared to be well accommodated into the hydrophobic pocket of Akt2 lined by Phe310, Phe359, L317 and Pro314, thus mimicking the packing of the +1 Phe of the GSK3 peptide in the active site of the kinase (Fig 7). A similar accommodation of an arginine residue of RS-containing peptides, including LBR RS-peptides (consensus position 3), into a hydrophobic pocket of the so called docking groove, has been observed in the case of SRPK1 [4]. The optimal side chain packing of the +1 arginines into the Akt active site might contribute to the correct positioning of the preceding serines (S78, S80, S82), in close proximity to the ATP γ-phosphate (Fig 7 and Table 1). In line with this hypothesis, Arg was found to be quite frequent at the +1 position in a number of *in vitro* Akt substrates [43]. Then again, in the case of the Akt2/LBR-S80 model, Arg83 at position +3 appeared to compete the +1 Arg for a π-stacking interaction with the phenyl ring of Phe310 of Akt2 (Fig 7). The optimal positioning of Ser80 and Ser82 relative to the ATP γ-phosphate (distance < 4Å) (Fig 7 and Table 1), in conjunction with the low B-factors of their hydroxyl oxygen (Table 1) and of the backbone atoms (blue in Fig 7) of the LBR-S80 and LBR-S82 peptides around their phosphosites, which compare to those of the GSK3 peptide substrate in the case of the ATP/MG-bound complexes (Fig 6, right panel), is in perfect agreement with the observed high efficiency of these sites to act as optimal Akt substrates (Fig 4, right panel).

On the other hand, the inability of peptide R2 to get phosphorylated (Fig 3C) and of Ser84 to get as efficiently phosphorylated as Ser80 and Ser82 (Fig 4, right panel), might be due to the presence of proline at position +1, as already mentioned above. Indeed, as predicted by the final 3D-model of the Akt2/LBR-S84 complex, the +1 Pro (P85) caused a local change of the peptide backbone geometry resulting to an offset of the preceding peptide positions, which in turn, forced the adjacent potential phosphosite (S84) away from the ATP γ-phosphate (distance > 6 Å; Table 1). This observation, in conjunction with the high mobility (high B-factor) of the Ser84 hydroxyl oxygen (Table 1) and the high flexibility of the LBR-S84 peptide around the phosphosite (as indicated by high fluctuations of the corresponding backbone atoms shown in Fig 6 and by the high B-factors of the equivalent peptide atoms colored in red in Fig 7), may provide a possible explanation for the significantly lower efficiency of Ser84 and consequently the inability of the R2 peptide (Fig 3C), to act as an Akt substrate. In addition, and in further support to the incompatibility of Pro at position +1 of Akt substrates, the misalignment of Pro85 of the LBR-S84 peptide appeared to be dictated by Pro314: a stacking of their side-chains was observed in the final 3D-model of the ATP/MG-bound complex (Fig 7; Akt2/LBR-S84). Pro314 corresponds to +1 subsite specificity determinant 15 in Ser/Thr kinases [44]. Interestingly and in line with the mandatory non-Pro specificity at +1 of Akts [38], a leucine instead of a proline at this position appears to be necessary for the Pro specificity at +1 observed in the case of CDKs and MAP kinases [44].

## Conclusions

In this study, we used a combination of biochemical approaches and 3D-modeling followed by MD simulations to shed light on the phosphorylation of the RS domain of Lamin B Receptor by activated Akt.

Our *in vitro* phosphorylation results clearly show that Akt1 and Akt2 directly target the LBR RS domain with distinct specificity than SRPK1. More specifically, Akt kinases phosphorylate mainly Ser80 and Ser82 and to a lesser extent Ser84, whereas all serines of the RS domain represent more or less equal phosphoacceptor sites for SRPK1. Another interesting finding of our study is that Ser78 represents a weak but yet a recognizable site by Akt, despite the absence of the Akt consensus arginine at motif position -5. In line with the experimental results, our *in silico* studies suggest that short LBR RS-containing peptides bearing phosphosites either at Ser80 or Ser82 can act, at least, as optimal Akt substrates as GSK3 (a well-known substrate of Akt), since the corresponding peptides can be accommodated into the active site of Akt2 in a similar way. On the other hand, as revealed by our MD simulations, short peptides with phosphosites at Ser78 and Ser84 (lacking -5 Akt motif position and with a proline residue at +1, respectively) exhibit a much more dynamic behavior into the active site of Akt2 and this conformational flexibility, which extends to the region around their phosphosites, may account for the lower efficiency of these serines as Akt substrates.

Overall, in this study we provide evidence that, unlike in the case of SRPK1, both the region N-terminal the phosphosite as well as the adjacent to the phosphoserines site +1, are important for recognition and phosphorylation of LBR RS-peptides by Akt, with the latter substrate position being compatible with the arginine residues of RS-repeats. As it is known that phosphorylation of the RS domain of LBR results in the detachment of peripheral heterochromatin from the inner nuclear membrane [2], we propose that the concerted action of these kinases may modulate the extent of phosphorylation of this domain, thus representing a fine-tuning mechanism for regulating gene expression. Moreover, our work paves the way for further research on the relationship between the structural dynamics of Akt and its catalytic mechanism.

## Supporting Information

S1 Fig Initial 3D-models of LBR-peptides. The Akt2-bound form of 10mer LBR peptides (Fig 5A) modeled in this study and the known structure of the Akt2-bound GSK3-peptide (from the crystal structure, 1O6K) [26], used as the modeling template (see also Fig 5).
(TIF)

S2 Fig Monitoring of RMSD values along the multiple MD trajectories. Root-mean-square deviations of all backbone atoms referenced to their initial positions along the multiple MD trajectories of the (left) binary and (right) ternary complexes simulated in this study.
(TIF)

S3 Fig Sequence similarity between human Akt1 and Akt2. (A) Sequence alignment of the kinase domains (region determined in the Akt2 crystal structure, 1O6K) [26] of human Akt1 and Akt2. The amino acid numbering is according to the Akt2 sequence. Open and red shaded boxes correspond to similarities and identities, respectively. Secondary structure elements shown on the top row are extracted from the 1O6K crystal structure. Residues involved in substrate binding in the case of Akt2 [26] and in other Ser/Thr kinases [44] are indicated by asterisks. This part of the figure was produced using ESPript utility [46]. (B) Surface representation of the substrate peptide binding region of Akt2 (in the 1O6K crystal structure) colored according to sequence conservation between Akt1 and Akt2: green for invariant and orange for similar residues. The GSK3 peptide is shown as sticks. Substrate peptide positions are numbered as in Fig 5A. Akt2 key residues involved in GSK3-peptide binding [26] are indicated with white labels. This part of the figure was rendered using PyMOL.
(TIF)

S4 Fig Graph of phosphorylation efficiency of LBR serine residues versus B-factors of corresponding hydroxyl oxygens. The phosphorylation efficiency of potential phosphosites of the LBR RS-domain relative to GST-LBRNt(62–92) was deduced from Fig 4, right panel (i.e. 100 minus residual phosphorylation of each phosphosite following mutation). The B-factor values of the corresponding hydroxyl oxygen are as in Table 1.
(TIF)

S1 Table Details of the multiple MD models of the simulated binary complexes. RMSD values of backbone atoms referenced to related initial structures and corresponding simulation time of the representative structure (see “Materials & Methods”) of each MD simulation of the binary complexes shown in S2 Fig (left panel).
(PDF)

S2 Table Details of the multiple MD models of the simulated ternary complexes. RMSD values of backbone atoms referenced to related initial structures and corresponding simulation time of the representative structure (see “Materials & Methods”) of each MD trajectory of the ternary complexes shown in S2 Fig (right panel).
(PDF)
