# Supplementary figures and images for: SRPK1 and Akt Protein Kinases Phosphorylate the RS Domain of Lamin B Receptor with Distinct Specificity: A Combined Biochemical and In Silico Approach
Source: PLoS One. 2016 Apr 22;11(4):e0154198. doi: 10.1371/journal.pone.0154198 (PMC4841541; doi:10.1371/journal.pone.0154198)

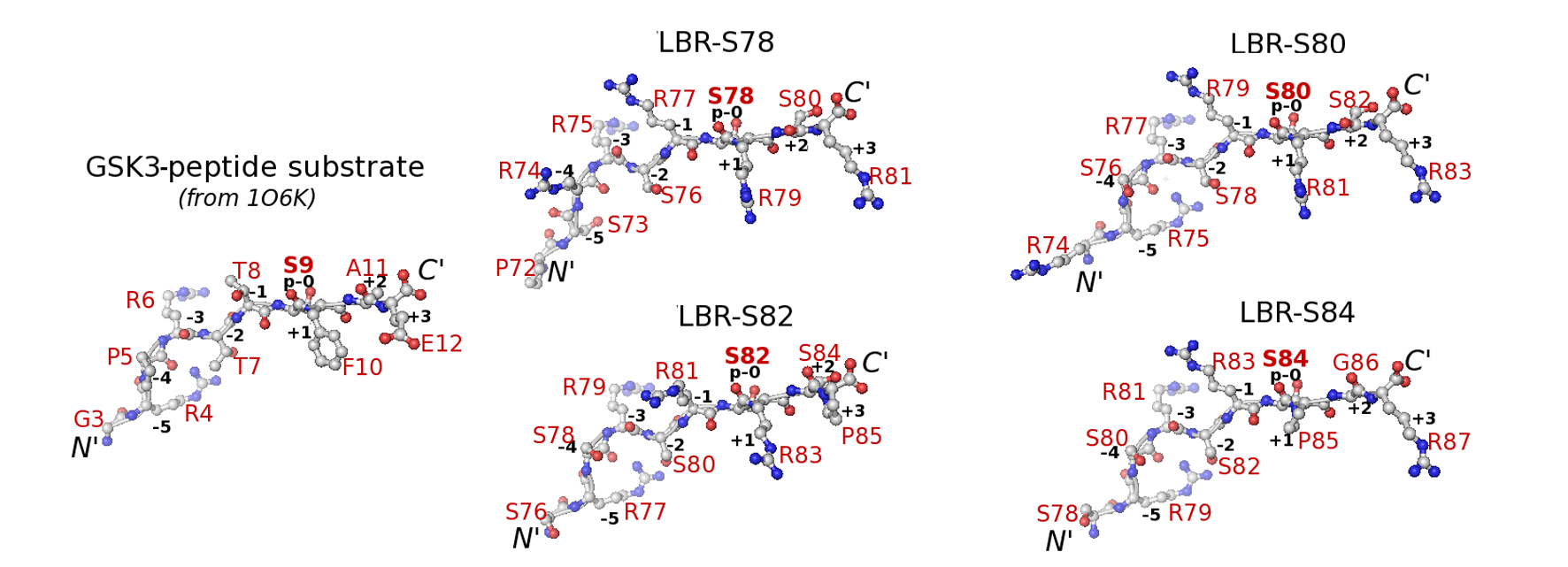

Supplement: S1 Fig — The Akt2-bound form of 10mer LBR peptides (Fig 5A) modeled in this study and the known structure of the Akt2-bound GSK3-peptide (from the crystal structure, 1O6K) [26], used as the modeling template (see also Fig 5). (TIF) [file pone.0154198.s001.tif]

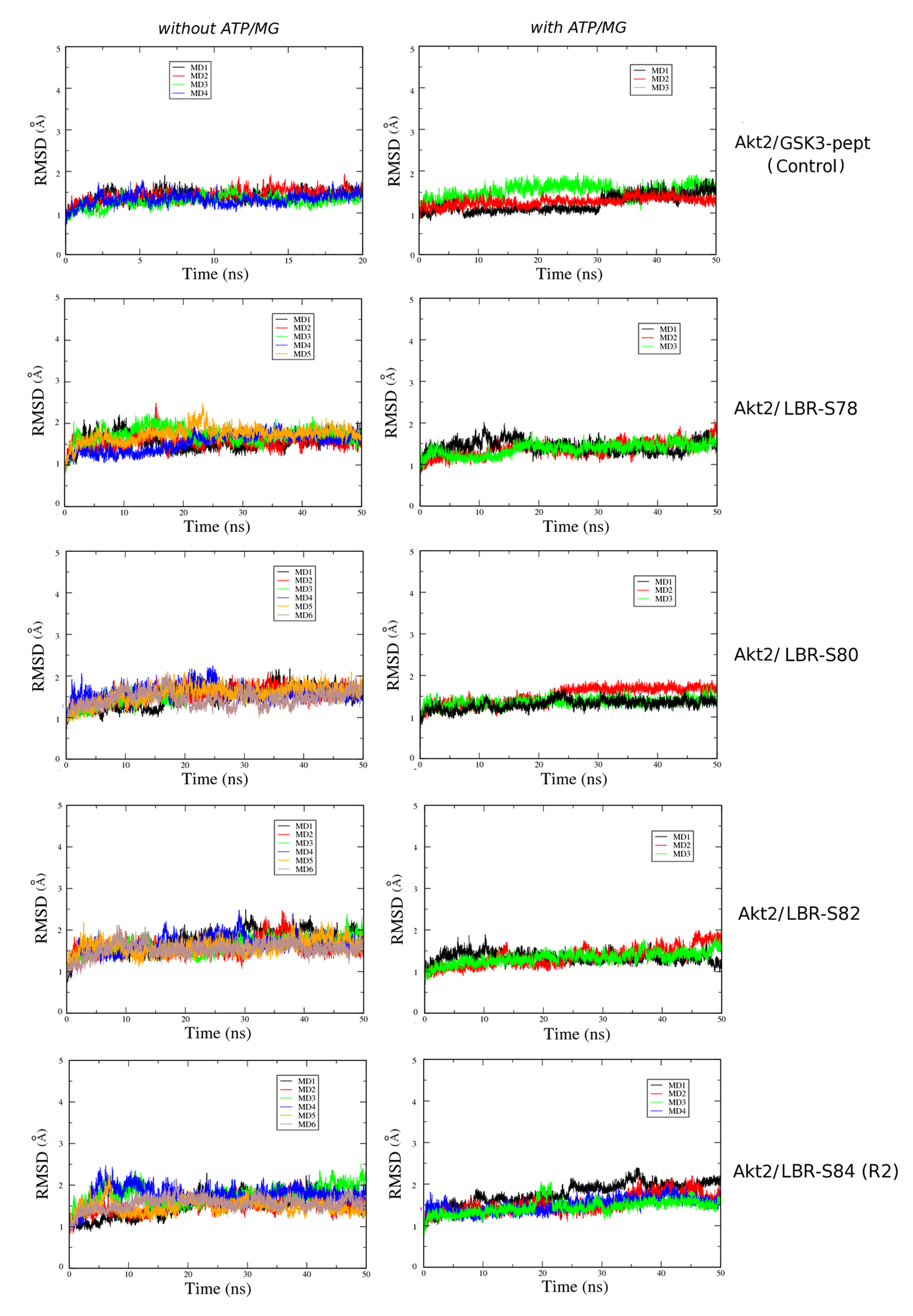

Supplement: S2 Fig — Root-mean-square deviations of all backbone atoms referenced to their initial positions along the multiple MD trajectories of the (left) binary and (right) ternary complexes simulated in this study. (TIF) [file pone.0154198.s002.tif]

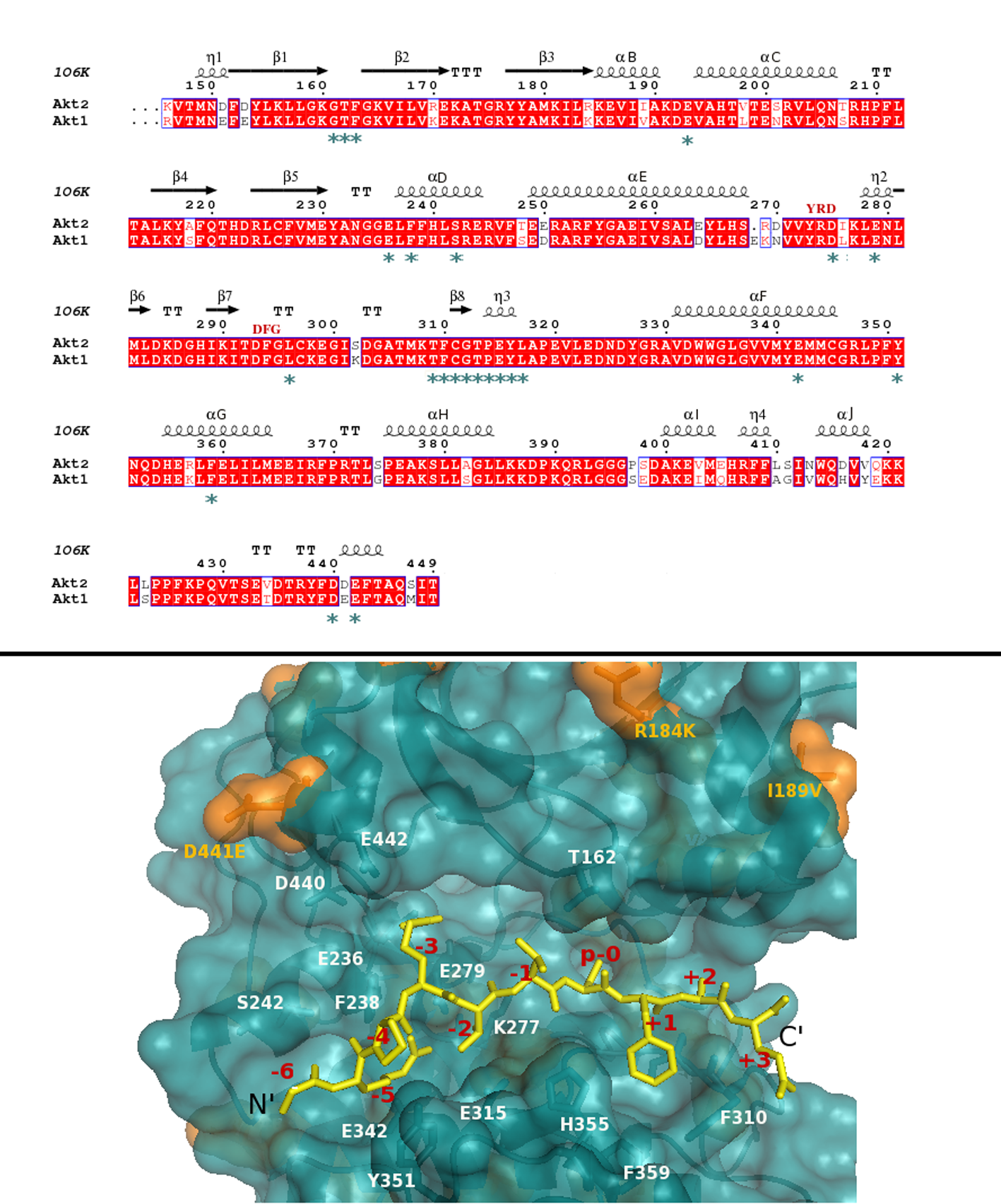

Supplement: S3 Fig — (A) Sequence alignment of the kinase domains (region determined in the Akt2 crystal structure, 1O6K) [26] of human Akt1 and Akt2. The amino acid numbering is according to the Akt2 sequence. Open and red shaded boxes correspond to similarities and identities, respectively. Secondary structure elements shown on the top row are extracted from the 1O6K crystal structure. Residues involved in substrate binding in the case of Akt2 [26] and in other Ser/Thr kinases [44] are indicated by asterisks. This part of the figure was produced using ESPript utility [46]. (B) Surface representation of the substrate peptide binding region of Akt2 (in the 1O6K crystal structure) colored according to sequence conservation between Akt1 and Akt2: green for invariant and orange for similar residues. The GSK3 peptide is shown as sticks. Substrate peptide positions are numbered as in Fig 5A. Akt2 key residues involved in GSK3-peptide binding [26] are indicated with white labels. This part of the figure was rendered using PyMOL. (TIF) [file pone.0154198.s003.tif]

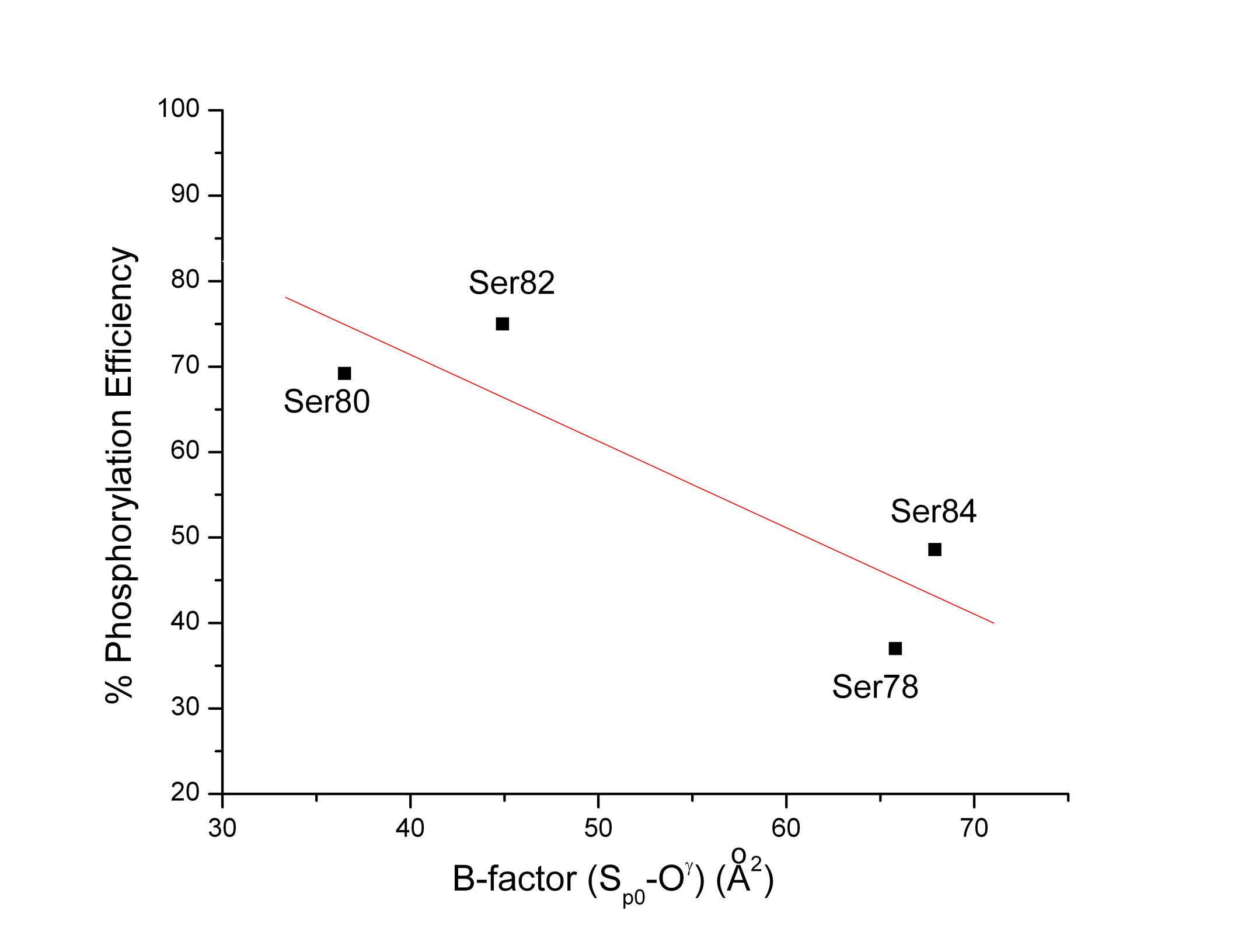

Supplement: S4 Fig — The phosphorylation efficiency of potential phosphosites of the LBR RS-domain relative to GST-LBRNt(62–92) was deduced from Fig 4, right panel (i.e. 100 minus residual phosphorylation of each phosphosite following mutation). The B-factor values of the corresponding hydroxyl oxygen are as in Table 1. (TIF) [file pone.0154198.s004.tif]
