# Supplementary material for: SRPK1 and Akt Protein Kinases Phosphorylate the RS Domain of Lamin B Receptor with Distinct Specificity: A Combined Biochemical and In Silico Approach
Source: PLoS One. 2016 Apr 22;11(4):e0154198. doi: 10.1371/journal.pone.0154198 (PMC4841541; doi:10.1371/journal.pone.0154198)
Supplement: S1 Table — RMSD values of backbone atoms referenced to related initial structures and corresponding simulation time of the representative structure (see “Materials & Methods”) of each MD simulation of the binary complexes shown in S2 Fig (left panel). (PDF) [file pone.0154198.s005.pdf]

Simulated System / MD Run	RMSD (Å) (Snapshot time /Total simulation time)				
	Akt2/GSK3-pept	Akt2/LBR-S78	Akt2/LBR-S80	Akt2/LBR-S82	Akt2/LBR-S84
MD1	1.33 (18.02 /20 ns)	1.55 (45.56 /50ns)	1.64 (42.47 /50ns)	1.84 (46.81 /50 ns)	1.56 (47.10 /50ns)
MD2	1.43 (15.68 ns)	1.48 (46.22 ns)	1.55 (45.46 ns)	1.47 (44.14 ns)	1.37 (41.62 ns)
MD3	1.17 (17.97 ns)	1.56 (46.16 ns)	1.64 (45.94 ns)	1.70 (43.01 ns)	1.89 (46.92 ns)
MD4	1.37 (16.67 ns)	1.57 (43.51 ns)	1.49 (48.67 ns)	1.71 (43.90 ns)	1.72 (45.83 ns)
MD5		1.66 (43.73 ns)	1.67 (41.71 ns)	1.66 (46.38 ns)	1.36 (46.06 ns)
MD6			1.39 (42.74 ns)	1.54 (42.08 ns)	1.50 (45.14 ns)
