# Supplementary material for: SRPK1 and Akt Protein Kinases Phosphorylate the RS Domain of Lamin B Receptor with Distinct Specificity: A Combined Biochemical and In Silico Approach
Source: PLoS One. 2016 Apr 22;11(4):e0154198. doi: 10.1371/journal.pone.0154198 (PMC4841541; doi:10.1371/journal.pone.0154198)
Supplement: S2 Table — RMSD values of backbone atoms referenced to related initial structures and corresponding simulation time of the representative structure (see “Materials & Methods”) of each MD trajectory of the ternary complexes shown in S2 Fig (right panel). (PDF) [file pone.0154198.s006.pdf]

	RMSD (Å) (Snapshot time /Total simulation time)				
Simulated System / MD Run	Akt2/GSK3-pept · ATP/MG	Akt2/LBR-S78 · ATP/MG	Akt2/LBR-S80 · ATP/MG	Akt2/LBR-S82 · ATP/MG	Akt2/LBR-S84 · ATP/MG
MD1	1.35 (42.53 /50ns)	1.28 (43.86 /50ns)	1.27 (42.49 /50ns)	1.29 (43.66 /50ns)	1.89 (45.70 /50ns)
MD2	1.25 (46.78 ns)	1.53 (45.39 ns)	1.67 (47.43 ns)	1.69 (46.01 ns)	1.77 (45.09 ns)
MD3	1.61 (47.72 ns)	1.37 (49.43 ns)	1.33 (41.99 ns)	1.37 (47.42 ns)	1.44 (48.46 ns)
MD4					1.51 (45.76 ns)
